# Post-Transformation IGHV-IGHD-IGHJ Mutations in Chronic Lymphocytic Leukemia B Cells: Implications for Mutational Mechanisms and Impact on Clinical Course

**DOI:** 10.3389/fonc.2021.640731

**Published:** 2021-05-25

**Authors:** Davide Bagnara, Catherine Tang, Jennifer R. Brown, Siddha Kasar, Stacey Fernandes, Monica Colombo, Stefano Vergani, Andrea N. Mazzarello, Fabio Ghiotto, Silvia Bruno, Fortunato Morabito, Kanti R. Rai, Jonathan E. Kolitz, Jacqueline C. Barrientos, Steven L. Allen, Franco Fais, Matthew D. Scharff, Thomas MacCarthy, Nicholas Chiorazzi

**Affiliations:** ^1^ The Feinstein Institutes for Medical Research, Institute for Molecular Medicine, Northwell Health, Manhasset, NY, United States; ^2^ Department of Experimental Medicine, University of Genoa, Genoa, Italy; ^3^ Department of Applied Mathematics and Statistics, State University of New York at Stony Brook, Stony Brook, NY, United States; ^4^ Chronic Lymphocytic Leukemia Center, Department of Medical Oncology, Dana-Farber Cancer Institute, Harvard Medical School, Boston, MA, United States; ^5^ Molecular Pathology, IRCCS Ospedale Policlinico San Martino, Genoa, Italy; ^6^ Biotechnology Research Unit, Azienda Ospedaliera of Cosenza, Cosenza, Italy; ^7^ Hematology and Bone Marrow Transplant Unit, Hemato-Oncology Department, Augusta Victoria Hospital, East Jerusalem, Israel; ^8^ Department of Medicine, Zucker School of Medicine at Hofstra/Northwell, Hempstead, NY, United States; ^9^ Department of Cell Biology, Albert Einstein College of Medicine, Bronx, NY, United States

**Keywords:** chronic lymphocytic leukemia, immunoglobulin genes, somatic mutations, mutation mechanisms, activation-induced deaminase

## Abstract

Analyses of IGHV gene mutations in chronic lymphocytic leukemia (CLL) have had a major impact on the prognostication and treatment of this disease. A hallmark of IGHV-mutation status is that it very rarely changes clonally over time. Nevertheless, targeted and deep DNA sequencing of IGHV-IGHD-IGHJ regions has revealed intraclonal heterogeneity. We used a DNA sequencing approach that achieves considerable depth and minimizes artefacts and amplification bias to identify IGHV-IGHD-IGHJ subclones in patients with prolonged temporal follow-up. Our findings extend previous studies, revealing intraclonal IGHV-IGHD-IGHJ diversification in almost all CLL clones. Also, they indicate that some subclones with additional IGHV-IGHD-IGHJ mutations can become a large fraction of the leukemic burden, reaching numerical criteria for monoclonal B-cell lymphocytosis. Notably, the occurrence and complexity of post-transformation IGHV-IGHD-IGHJ heterogeneity and the expansion of diversified subclones are similar among U-CLL and M-CLL patients. The molecular characteristics of the mutations present in the parental, clinically dominant CLL clone (CDC) differed from those developing post-transformation (post-CDC). Post-CDC mutations exhibit significantly lower fractions of mutations bearing signatures of activation induced deaminase (AID) and of error-prone repair by Polη, and most of the mutations were not ascribable to those enzymes. Additionally, post-CDC mutations displayed a lower percentage of nucleotide transitions compared with transversions that was also not like the action of AID. Finally, the post-CDC mutations led to significantly lower ratios of replacement to silent mutations in VH CDRs and higher ratios in VH FRs, distributions different from mutations found in normal B-cell subsets undergoing an AID-mediated process. Based on these findings, we propose that post-transformation mutations in CLL cells either reflect a dysfunctional standard somatic mutational process or point to the action of another mutational process not previously associated with IG V gene loci. If the former option is the case, post-CDC mutations could lead to a lesser dependence on antigen dependent BCR signaling and potentially a greater influence of off-target, non-IG genomic mutations. Alternatively, the latter activity could add a new stimulatory survival/growth advantage mediated by the BCR through structurally altered FRs, such as that occurring by superantigen binding and stimulation.

## Introduction

Chronic lymphocytic leukemia is a relatively common, yet broadly divergent disease (1, 2). Some patients survive for decades without therapy, while others succumb soon after diagnosis. At the molecular level, this heterogeneity is also prominent and linked to the clinical condition. Most relevant, the level of mutations in the IGHV genes expressed by leukemic clones segregate CLL patients into two groups (3), and patients fitting into these categories have divergent clinical courses and outcomes (4, 5). In general, those patients with leukemic clones expressing an IGHV with somatic mutations exceeding a defined threshold (IGHV-mutated, M-CLL) have better prognoses, whereas those patients whose leukemic clones express an IGHV without or with minimal numbers of mutations (IGHV-unmutated, U-CLL) experience more severe disease. For this reason, it is currently recommended that IGHV-mutation status be determined at the time of diagnosis to help physicians identify and more closely follow patients at greater risk (6).

The IGHV-mutation status used in the clinic is usually determined by standard Sanger DNA sequencing (7), and rarely changes at the clonal level over time. Because the sensitivity of the Sanger sequencing approach is low, the likelihood of detecting minor intraclonal variants differing in IGHV mutations is small and can be inconclusive. Despite this, by sequencing relatively large numbers of IGHV-IGHD-IGHJ molecular clones across a panel of patients (8, 9) or within a distinct stereotyped subset (10) or in single cells (11), intraclonal IGHV-IGHD-IGHJ heterogeneity has been found. This was subsequently confirmed when next generation, deep DNA sequencing (NGS) became available (12).

In addition to corroborating that intraclonal V-region diversity exists, the latter study highlighted several important considerations that could be incorporated into future studies of the process (12). These emphasized that the DNA sequencing approach employed needs to have considerable sensitivity (“sequencing depth”) and contain sufficient safeguards to insure accuracy (e.g., avoidance of PCR and DNA sequencing artifacts). This was seen as essential since the number of cells with unique mutations that develop in the IGHV-IGHD-IGHJ sequence might be small. The authors also indicated the necessity to study a patient cohort with a large number of patients differing in IGHV-mutation status to determine if the generation of intraclonal IGHV-IGHD-IGHJ variants differed between U-CLL and M-CLL clones. Additionally, there should be enough unique reads to determine if the mutations identified bear the characteristics of somatic hypermutation (SHM) caused by activation-induced deaminase (AID) (13) and error prone mismatch repair (14), and if there is in silico evidence for antigen-selection of the sequence variants. Finally, the CLL cases studied should have an adequate period of follow-up to establish if the intraclonal process links with clinical course and outcome.

In light of this background, we have addressed the frequency and degree of intraclonal IGHV-IGHD-IGHJ heterogeneity in CLL, the extent that the subclones exhibiting this contribute to the clonal burden, the characteristics of the post-transformation mutations, and the association of intraclonal IGHV-IGHD-IGHJ diversity with clinical outcome. To tackle these issues, we used an NGS approach of considerable depth that incorporates Unique Molecular Identifiers (UMIs) to minimize technical artefacts and PCR amplification bias (15) to study the IGHV-IGHD-IGHJ regions in CLL clones from a cohort of well characterized, untreated patients, for whom clinical and laboratory features and temporal follow-up were available.

Our study extends previous findings and documents that: the intraclonal IGHV-IGHD-IGHJ diversification process is virtually ubiquitous and takes place with equal frequency and extent in both U-CLL and M-CLL patients; certain subclones expressing new IGHV-IGHD-IGHJ mutations can be significantly expanded *in vivo*; the fraction of mutations bearing SHM marks is considerably less than in the parental clone and these are inconsistent with selection for enhanced antigen binding; and finally some of the features of the post-transformation mutations might be useful adjuncts to the standard U-CLL vs. M-CLL IGHV mutation status prognostic approach.

## Methods

### Samples

This study was approved by the Institutional Review Boards of Northwell Health and of Dana Farber Cancer Center. Written informed consent was obtained in accordance with the Declaration of Helsinki. PBMCs were isolated from untreated CLL patients by density gradient centrifugation (Ficoll, GE Healthcare), suspended in in RPMI1640 medium with 50% FBS, and stored in liquid nitrogen until used.

### Cell Separation

PBMCs from 45 CLL samples collected at Northwell Health were incubated with V500 anti-CD19 (BD Biosciences) and PE-cy7 anti-CD5 (Invitrogen). After excluding dead cells using Sytox Blue (ThermoFisher), live CD19^+^CD5^+^ B cells were sorted directly into tubes containing Dynabeads Oligo(dT) (ThermoFisher) lysis buffer and stored at −80°C.

For the 15 CLL PBMC samples collected at Dana Farber Cancer Institute, B cells were isolated using RosetteSep negative selection prior to density gradient centrifugation if the white blood cell count was less than 25,000/ml or the absolute lymphocyte count was less than 20,000/ml.

### Library Preparation and Sequencing

Library preparation and sequencing were performed as described (15) and illustrated in **Supplemental Figure S1**. Briefly, mRNA was isolated with Dynabeads Oligo (dT) in 200 µl PCR tubes as instructed for the Dynabeads macknowledRNA DIRECT Micro Kit (ThermoFisher). cDNA was synthesized in solid phase using SuperScript III Enzyme (ThermoFisher). Second strand synthesis was performed with a mix of IGHV leader sequence specific primers, and Unique Molecular Identifiers (UMIs) and Illumina adaptors were introduced at this point. Purified ds-cDNA was used as template to amplify, by a semi-nested approach, the entire IGHV-IGHD-IGHJ rearrangement and a portion of the constant region sufficient to identify IgM, IgG and IgA.

The product was quantified with Qubit (ThermoFisher) and 1–10 ng was used to add the Illumina Index with the Nextera XT kit (Illumina). Pooled libraries were sequenced with MiSeq Illumina (v3.2 × 300 kit, Illumina MS-102-3003). Raw data are available at SRA (BioProject ID PRJNA673787-https://www.ncbi.nlm.nih.gov/Traces/study/?acc=PRJNA673787&o=acc_s%3Aa).

### Bioinformatic and Statistical Analyses

Processing of raw reads was performed with pRESTO (16). Processed sequences were submitted to IMGT/HighV-QUEST. Data handling was carried out using ChangeO (17) and R. Prism 8 and R were used for statistical analyses. All box plots in the figures represent medians and quartiles. Non-productive IGHV-IGHD-IGHJ rearrangements were excluded from all analyses, except those dealing with mutation targeting and selection.

A Cox proportional hazards regression analysis was used to test mutation, complexity and their interaction (mutation × complexity) in the model. The “interaction” between mutation and complexity measures whether the effect of complexity in the *IGHV*-mutated group is the same or different from its effect in the *IGHV*-unmutated group. To visualize these results, the Kaplan–Meier curves for TTFT and survival were generated for the four groups (M-CLL^High^, M-CLL^Low^, U-CLL^High^, U-CLL^Low^). Pairwise multiple comparisons were carried out for differences among the four groups without adjustment for multiple testing. All results were considered statistically significant if *P <*0.05.

### Subclonal Expansion Sensitivity Threshold

For 27 samples, we sequenced four or eight replicates, each containing 25,000 cells from the same blood draw (**Supplemental Table S1**); replicates were used to calculate the sensitivity threshold to identify expanded subclonal variants. The approach is based on the principle that, if a subclonal variant is repeatedly observed in several sequencing replicates, it derives from expanded B cells with the identical IGHV-IGHD-IGHJ sequence. **Supplemental Figure S2** shows the frequency distribution of the subclones observed in at least ¾ of the replicates. We chose the 25th percentile (0.008%) of the distribution as a conservative threshold to identify expanded variants. This threshold corresponds to two cells in a 25,000 cell replicate; this number is possible because, as described (15), our library preparation and sequencing approach exhaustively determines the repertoire of all starting B cells. The calculated sensitivity threshold for subclonal amplification was then applied to all samples analyzed.

### Clonal Inference and Phylogeny

For each sample, we identified the CLL clonal family with ChangeO (17), using a distance threshold of 0.07. Since the repertoire diversity of CD5^+^ B cells in a CLL patient is very low, we were able to perform a “manual” assessment of the clonal attribution to correct possible false positives, false negatives, and PCR artifacts such as chimeras that might have escaped error correction. The phylogenetic tree for each CLL clonal family was inferred with the R package Alakazam (18), and the trees were plotted with igraph (Csardi G, Nepusz T. 2006. The igraph software package for complex network research. InterJournal, Complex Systems, 1695. https://igraph.org).

### Mutation Targeting Analysis

We used the dominant, consensus IGHV-IGHD-IGHJ sequence found for each patient as the clinically-dominant clone (CDC), and used this as the reference for all variant mutation calls (“post-CDC mutations”). In order to evaluate whether mutations in the post-CDC sequences were targeted to hotspots or not, we assumed that the mutations occurred in the CDC sequence background. For each sample and category, we added up the mutations from each unique sequence, then considered the context in which they occurred (hotspot or non-hotspot). To evaluate mutational enrichment to AID (WRC/GYW) hotspots, we compared the ratio (h:n) of mutations targeting hotspots (h) to non-hotspots (n) to the expected proportion (number of hotspots/number of G:C sites) using a binomial test (19). Benjamini–Hochberg correction was then applied at the sample level. Using the same method, we also considered hotspots for APOBEC3A/B (TC/GA) and Polη (WA/TW), where the expected proportion used A:T sites in denominator); these are similar to Signatures 2 and 13 and to Signature 9, respectively, as defined in the Catalogue of Somatic Mutations in Cancer (COSMIC) database [Tate et al. (20)] (cancer.sanger.ac.uk/cosmic). Finally, we compared the observed mutations to those ascribed by the COSMIC database to Signatures 1 (“Aging signature”) and 5 (“Cancer signature”). Signature 1 is the result of an endogenous mutational process initiated by spontaneous deamination of 5-methylcytosine (N(C>T)G), and Signature 5 exhibits transcriptional strand bias for T > C substitutions at ApTpN context. One-sided tests were used to evaluate higher targeting to hotspots. Finally, those sequences representing a non-productive IGHV-IGHD-IGHJ rearrangement, which were present at a frequency of 0.008% (25th percentile of the intraclonal subclone distribution used to define subclonal amplification) and hence more likely derived from CDC subclones (n = 17), were used in mutation analysis as mutations that could not undergo antigen selection.

### Selection Analysis

The selection pressure on mutations in the VH Complementarity Determining Regions (CDRs) and Framework Regions (FRs) was calculated using the Bayesian estimation of Antigen-driven SELectIoN (BASELINe) (21, 22). This algorithm compares observed and expected mutations estimated with an Ig-specific targeting model of SHM and calculates the posterior probability density function represented in the plots.

## Results

### Approach

We carried out NGS of full length IGHV-IGHD-IGHJ rearrangements in 62 CLL patients to define intraclonal variants differing in cDNA sequences from those found in the CDC. We define the CDC as the overriding IGHV-IGHD-IGHJ sequence determined by Sanger sequencing of circulating CLL cells and confirmed by NGS. In some cases, additional subclones, unrelated to the CDC based on VH CDR3 sequence differences, were identified; these were excluded from the following analyses.

We used our sequencing methodology that employs Unique Molecular Identifiers (UMIs) to label each mRNA molecule and high sequencing depth, thereby improving error correction capacity, attainment of high quality sequences, and enhanced sequencing sensitivity (15) (**Supplemental Figure S1**). In addition, to minimize the artificial diversity inevitably arising from errors in DNA sequencing and PCR, here we have also included only those unique sequences that were observed with ≥3 distinct UMIs (corresponding to three different mRNA molecules sequenced) and that came from the consensus of ≥5 reads. Thus, this is a very high bar for error correction and filtering and further avoids misinterpretations based on inaccuracies or randomness. Using these cutoffs, on average for each sample 2.3 million reads met our filtering criteria (effective “sequencing depth” = 2,300,000×) carrying 0.4 million distinct UMIs representing 540 unique nucleotide sequences (intraclonal subclones) for an individual CLL IGHV-IGHD-IGHJ rearrangement. Details of each sample are provided in **Supplemental Table S1**. All frequencies reported below refer to the abundance of mRNA molecules sequenced as quantified by UMIs.

### Detection of Intraclonal IGHV-IGHD-IGHJ Rearrangement Variants in Patients With CLL

**Figure 1:**
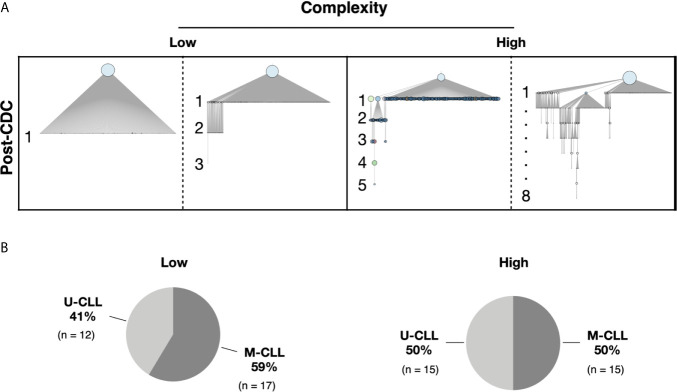
Complexity of intraclonal IGHV-D-J variants. **(A)** Examples of the degree of intraclonal diversity in CLL samples based on NGS sequencing data. The phylogenetic trees were inferred with the R package Alakazam and plotted with igraph (see *Methods*). The size of the CDC (light blue circles at top of each example) is proportional to its abundance within the entire leukemic clonal family. IGHV-D-J complexity is defined and represented by the number of distinct sequences downstream of the CDC. Each downstream sequence has all the mutations of the upstream sequence plus at least one additional. Low complexity contains one to three mutation-defined sequences downstream of the initial branch from the CDC. High complexity represents four or more unique sequences downstream of each initial branch, some involving intricate branching. **(B)** Pie graphs indicating the representation of U-CLL and M-CLL cases in the Low and High complexity categories.

We defined an “intraclonal IGHV-IGHD-IGHJ subclone” as an IGHV-IGHD-IGHJ sequence that is clonally-related to the CDC based on HCDR3 similarity and use of the same IGHV and IGHJ genes but differing from the CDC by at least one nucleotide somewhere in the entire IGHV-IGHD-IGHJ region. These clonally related progeny downstream of the CDC are hereafter referred to as post-CDC intraclonal subclones. Despite the stringent error correction and filtering of our approach, all but one patient sample (61/62, 98%) bore IGHV-IGHD-IGHJ rearrangements differing in sequence from the CDC. The one case in which significant variants were not detected had much lower sequencing depth compared with the average (194,000× vs. 2,300,000×). **Figure 1A** provides representative examples of the types of differences uncovered; these changes reflect not only the number of IGHV-IGHD-IGHJ mutations that developed post-transformation but also the complexity of that new mutational load, illustrated by the existence and extent of branching found in certain cases.

**Figure 2:**
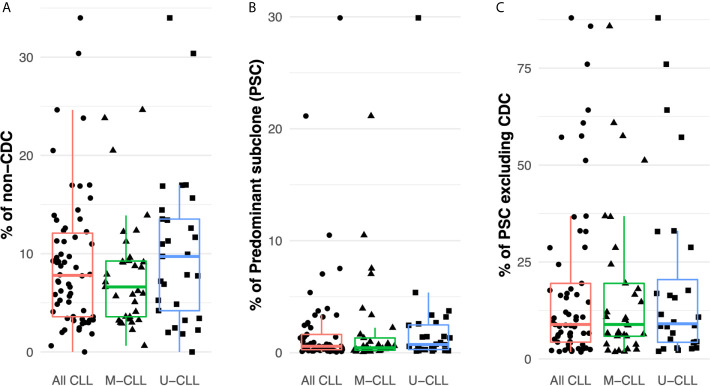
Intraclonal IGHV-D-J diversity is relatively common in CLL. **(A)** Fraction of the intraclonal IGHV-D-J variant sequences among the total number of mRNA molecules coding the CLL clonal family in the 61 patients examined. **(B)** Frequency of PSCs among the total number of mRNA molecules encoding the CLL clonal family. **(C)** Fraction of the subclonal IGHV-D-J variants that the PSCs represent after excluding the CDC sequences.

Considering all patient samples, the median frequency of IGHV-IGHD-IGHJ intraclonal subclones differing from the CDC was 8% (distribution 0–34%) (**Figure 2A**). The most abundant intraclonal subclone (“predominant subclone”, PSC) was defined by the percentage of the total defined IGHV-IGHD-IGHJ repertoire that the PSC represented. This was estimated to be on average 0.6%, ranging in frequency from 0 to 29% (**Figure 2B**). Additionally, we calculated the PSC frequency among only the members of each subclone whose sequences differed from that CDC. This ranged from 1.8–88% with a median of 9% of the variant fraction (**Figure 2C**).

Finally, after dividing the samples based on IGHV-mutation status, both M-CLL and U-CLL clones exhibited similar frequencies of intraclonal variants (**Figure 2A** **)**. Moreover, the percentage of the variants represented by the PSC was not significantly different between the two CLL subtypes (**Figure 2B**). When the CDC were excluded, the average frequency of unique sequences did not differ between M-CLL and U-CLL cases (**Figure 2C**).

In summary, IGHV-IGHD-IGHJ sequences differing from that of the CDC exist within virtually all CLL clones (~98% here). Additionally, when focusing on the PSCs, the ratio of these intraclonal variant sequences varies among patients. Although in most instances the ratio is relatively low, at times it can be sizeable. Notably, each finding is similar in M-CLL and U-CLL, indicating that these intraclonal phenomena occur in all CLL clones, regardless of IGHV-mutation status.

### Expansion of Subclones Downstream of the CDC

**Figure 3:**
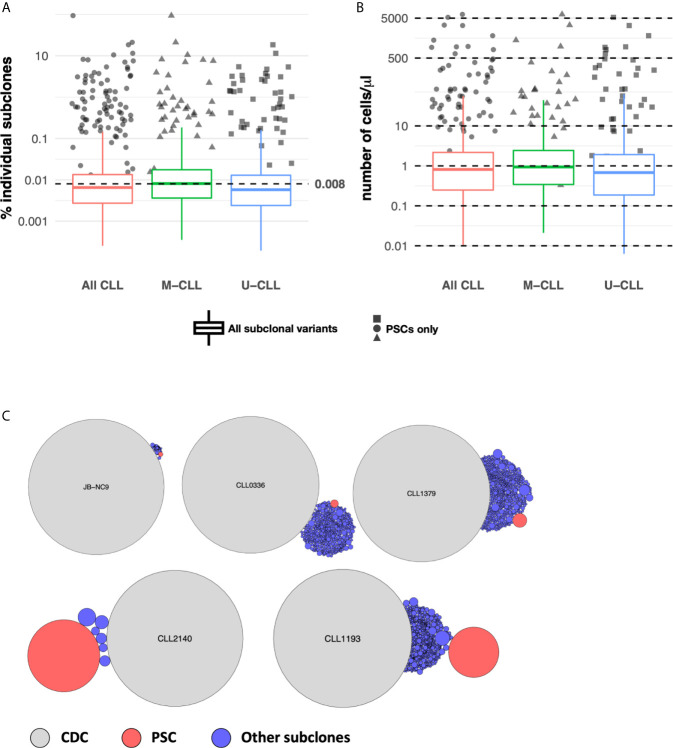
Subclonal variant cell counts. **(A)** PSCs are numerically expanded. Box plots indicate the percentage distribution of all post-CDC subclones. The solid colored dots represent the percentages of individual PSCs. The dotted horizontal line indicates the 0.008% sensitivity threshold used to define subclonal expansion (see *Methods*). **(B)** Absolute numbers of distinct IGHV-D-J subclones per ul of individual patient blood samples estimated from sequencing data, flow cytometry, and absolute blood lymphocyte counts. Box plots and solid colored dots as in **(A)**. **(C)** Representative examples of the relative numbers of the post-CDC subclones (blue circles) and the PSCs (red circles) in relation to the CDC of five samples analyzed in this study.

Next we determined the relative sizes of the CLL subclones by assigning a sensitivity threshold to minimize the likelihood of random sampling of sequences carried by expanded B cells. This was possible because when CLL cells were sorted, for 28 samples at least four aliquots of 25,000 cells were collected (**Supplemental Table S1**), and each aliquot was sequenced separately. Using the data from these replicates, we calculated the frequency distribution of the unique IGHV-IGHD-IGHJ sequences found in at least 75% of the aliquots (*Methods* and **Supplemental Figure S2**), and used the 25th percentile of the distribution (0.008%) as a cut-off for B-cell expansion. **Figure 3A** shows that all post-CDC PSCs are well above this cut-off.

Next, using the absolute number of lymphocytes in each patient’s blood sample, we inferred the numerical size of individual subclones in the blood. This calculation revealed that almost all PSCs exceeded a count of 10 cells/µl, in comparison to the median count of the entire set of subclones which was 1.5 cells/µl (distribution 0.01–3,616 cells/µl; **Figure 3B** **).** **Figure 3C** illustrates representative examples of the calculated size of the PSC compared to the CDC and to the other IGHV-IGHD-IGHJ intraclonal subclones.

Thus, many of the intraclonal subclones, especially the most frequent fraction (PSC), are numerically expanded, although the relative sizes vary. Therefore, the PSCs appear to be “advantaged” in some way.

### Extent of Intraclonal Complexity Occurring Downstream of the CDC

The previous analyses were based solely on the presence or absence of IGHV-IGHD-IGHJ mismatches from the CDC sequence. Next, we divided the cases by the extent of intraclonal complexity/architecture using branching from the CDC. Fifty-nine of the samples were included in this analysis; for the three cases excluded, no subclones were found in one, and the software used could not construct phylogenetic intraclonal IGHV-IGHD-IGHJ trees for the other two.

To perform this analysis, we partitioned the samples into two complexity categories. The “Low” group contains one to three mutations downstream of the initial branch from the CDC (see **Figure 1A** for examples). The “High” group exhibited four or more unique sequences downstream of each initial branch (**Figure 1A**). Of the 59 patients, 29 fell into the Low category and 30 into the High category (**Figure 1B**). Notably, the U-CLL and M-CLL patients were relatively uniformly distributed based on complexity levels (Low complexity—12 M-CLL and 17 U-CLL; High complexity—15 M-CLL and 15 U-CLL; **Figure 1B**).

Thus, U-CLL and M-CLL clones display intraclonal differences at similar frequencies, and the presence and extent of post-CDC complexity are very similar among them as well.

### Characterization of Mechanisms Inducing and Repairing the Post-CDC IGHV-IGHD-IGHJ Mutations

Next, we analyzed the specific post-CDC IGHV-IGHD-IGHJ rearrangements for evidence that the mutations were the consequences of mutational mechanisms associated with SHM (targeting AID hotspots and avoiding AID coldspots [SYC: s (G/C; C/T; C) (23)] or bearing features of error prone repair by Polη. We also looked for mutational signatures found outside IG loci as listed in COSMIC v86 (20), focusing on Signatures 1 (Aging), 2 and 13 (APOBEC) and 5 (Cancer). The mutation signatures for these are listed in *Methods*.

**Figure 4:**
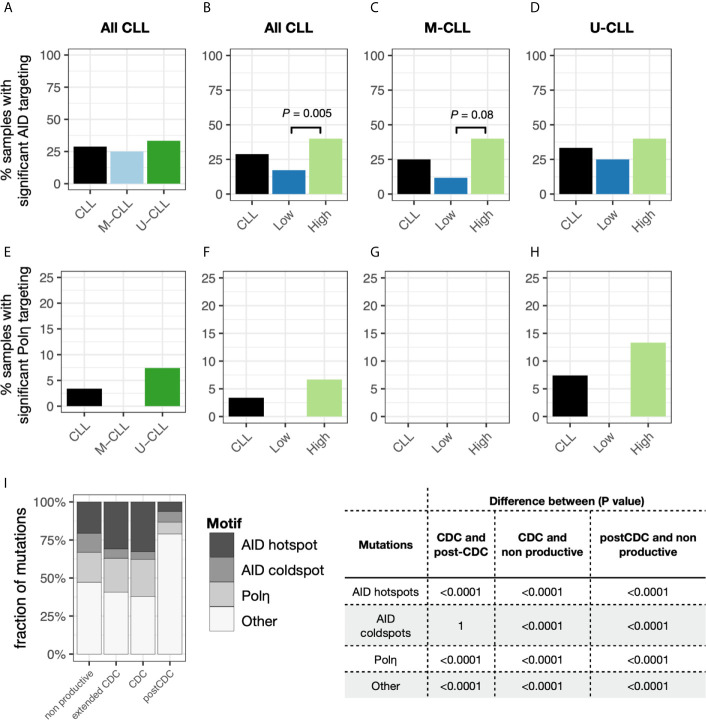
Relative targeting of mutational mechanisms. **(A–D)** Percentage of samples exhibiting significant targeting to AID hot spots in subclones, based on IGHV-mutation status **(A)**, IGHV-mutation status + level of IGHV-D-J complexity for all CLL samples **(B)** or for only M-CLL **(C)** or only U-CLL cases **(D)**. **(E–H)** Percentage of samples exhibiting significant features of Polη repair in subclones, based on IGHV-mutation status **(E)**, IGHV-mutation status + level of IGHV-D-J complexity for all CLL samples **(F)** or for only M-CLL **(G)** or only U-CLL cases **(H)**. **(I)** Fraction of post-CDC mutations attributable to the actions of AID at hot spots (red), AID at cold spots (green), of Polη repair (blue), or other/unclear (purple). For each motif type (AID hotspot, AID coldspot, Polη hotspot, all others), we calculated the fraction of mutations targeting the motif. Within each sample, this was done separately for the CDC, post-CDC, and non-productive sequences, giving a pair of fractions per sample. After calculating pairs for each sample, then CDC, post-CDC and non-productive sequences were compared across all samples using a paired t-test. Table to the right indicates the mean fractional differences of mutations between the CDC and the post-CDC settings in regard to mutations targeting the three motifs and not attributable to the any of the three.

Notably, although there were some sequences bearing APOBEC and the other COSMIC signatures, none of the samples as a whole bore significant features consistent with these mechanisms (not shown). As expected, AID and Polη signatures were identified. Twenty-nine percent of the samples exhibited significant AID targeting, with no significant differences between U-CLL and M-CLL (**Figure 4A**). When incorporating the degree of intraclonal IGHV-IGHD-IGHJ complexity into the analysis, samples with significantly more High complexity displayed significantly more AID targeting compared with Low complexity samples (**Figure 4B**), with M-CLL cases being the majority (**Figures 4C, D** **)**. When examining Polη-related mutations, many fewer samples exhibited significant targeting (3.4%) (**Figure 4E**), and all of these were from U-CLL cases with High complexity (**Figure 4H**). No M-CLL and no U-CLL with Low complexity exhibited significant Polη signatures; hence the findings were not obviously influenced by the degree of IGHV-IGHD-IGHJ complexity (**Figures 4F, G** **)**. So, the dominant identifiable mutational signature found in the post-CDC samples is AID-related. Nevertheless, this represented only ~25% of the samples.

Next, we carried out similar analyses to determine if the mutational targeting found in the parent CDCs was like that found in the post-CDC samples and how these compared with those in non-productive IGHV-IGHD-IGHJ rearrangements found within the same patients. Notably, for the non-productive sequences, AID mutations accounted for 33% of the CDC mutations (targeting hotspots: 21%; avoiding coldspots: 12%) and Polη for 20% (**Figure 4I**). For the parental CDC, AID mutations accounted for a significantly greater level (35%) (hotspot: 30%; coldspot: 5%) and Polη for 26% (**Figure 4I**). In our CDC cohort, 13 samples did not bear *IGHV* mutations. Therefore, to assure that these findings were not influenced by the number of samples analyzed (n = 49), we also used an extended database containing 2,084 CLL IGHV-IGHD-IGHJ CDC sequences. When employing the larger data, still no samples bore significant evidence for APOBEC and COSMIC Signatures 1 and 5.

Next, we compared the distribution of mutations in the non-productive rearrangements and in the CDC sequences with those in the post-CDC (**Figure 4I**). This illustrated a striking change in the attribution of mutations to SHM between the CDC and post-CDC periods. Specifically, there were highly significant decreases in targeting of AID hotspots and in mutations resembling Polη action in relation to both the non-productive and productive (CDC) rearrangements. Additionally, there were highly significant increases of mutations targeting AID coldspots. Consequently, there was a very significant increase in the fraction of mutations that could not be assigned to SHM (**Figure 4I**).

**Figure 5:**
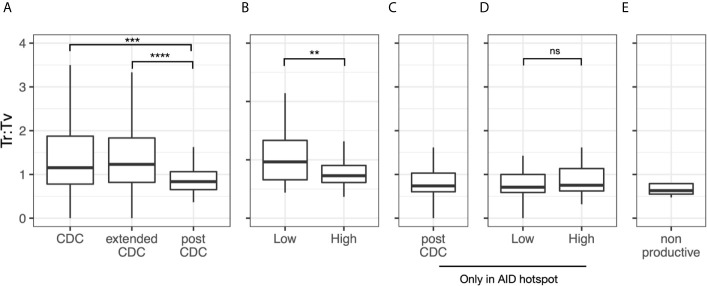
Transition to transversion ratios in the post-CDC mutations. Transition to transversion ratios (Ts : TV) analyzed for: **(A)** all post-CDC mutations, **(B)** all post-CDC mutations based on levels of complexity, **(C)** only those post-CDC mutations involving AID hotspots, **(D)** post-CDC mutations involving AID hotspots based on levels of complexity and **(E)** non-productive IGHV-IGHD-IGHJ rearrangements (**P ≤0.01, ***P ≤0.001, ****P ≤0.0001). NS, Not statistically significant.

Since AID induced mutations usually result in more base transitions (Ts) than transversions (Tv), usually ~1.5 Ts to 1 Tv (24–27), we analyzed this parameter as well. Overall, the median Ts : Tv for the post-CDC mutations grouped by sample was 0.80 (**Figure 5A**). Notably, this ratio was significantly less than that of the 49 CDC samples with IGHV mutations (median = 1.2; *P* = 0.0012) and of the IGHV-mutated cases in the extended CDC set (median = 1.3; *P <*0.0001). When analyzing the degree of IGHV-IGHD-IGHJ complexity to the post-CDC mutations, the High complexity samples had a significantly lower Ts : Tv than the Low complexity samples (0.79 *vs*. 1.09; P <0.01; **Figure 5B**). When analyzing those mutations occurring only at AID hot spots (**Figure 5C**), the Ts : Tv was 0.92, and no difference was seen based on IGHV-IGHD-IGHJ complexity (**Figure 5D**). Finally in the non-productive rearrangements, the Ts : Tv was 0.63 (**Figure 5E** **).** So, whereas the CDC samples bearing IGHV mutations have Ts : Tv similar to that expected, the post-CDC samples as well as the non-productive sequences have ratios very significantly lower than these.

In summary, these analyses suggest that the majority of mutations occurring in the post-CDC period are much less attributable to AID based SHM based on their type and targeting and the repair mechanisms employed than those mutations that developed in the CDC.

### Analyses of Selection Among the Post-CDC IGHV-IGHD-IGHJ Mutations

**Figure 6:**
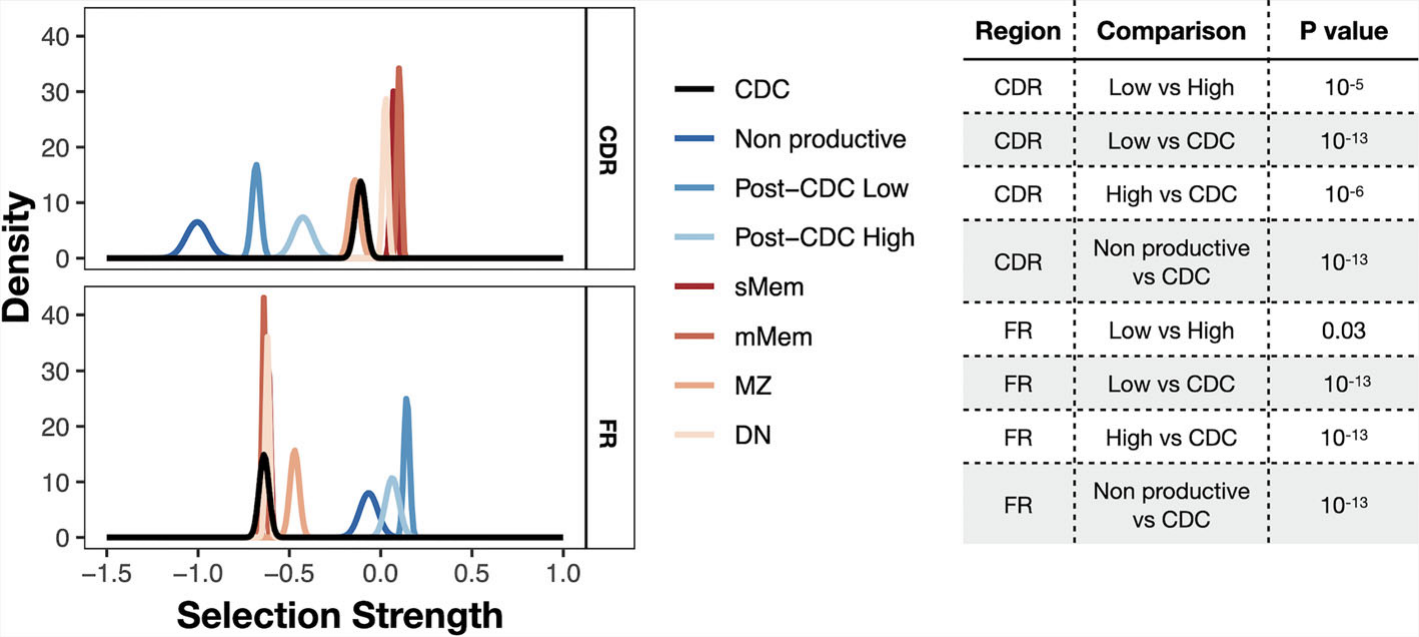
Selection strength on mutations in VH CDRs and the VH FWRs. The BASELINe program was used to calculate Selection Strength (Σ; see *Methods*) starting from observed and expected replacement to silent mutation ratios (R:S). Selection strength of mutations found in CDC and post-CDC productive and non-productive rearrangements based on complexity (post-CDC^High^ and post-CDC^Low^) as well as those of a series of normal human mature B cell subsets (IgM memory cells, mMem; isotype-class switched memory cells, sMem; marginal zone B cells (MZ); and IgD^-^CD27^−^ double negative B cells (DN). Σ indicates selection strength favoring selection for (right of 0) or against (left of 0) replacement mutations. Table to the right indicates the statistical analyses for selected differences between comparisons in the VH CDRs and VH FRs involving mutations detected in CDC and post-CDC productive and non-productive sequences divided based on post-IGHV-IGHD-IGHJ diversity into High and Low as described in **Figure 1**. All comparisons were significantly different except for the following: for CDR: CDC *vs*. MZ; mMem *vs*. sMem; and sMem *vs*. DN; for FR: post-CDC^High^ *vs*. post-CDC^Low^; post-CDC^High^ *vs*. non-Productive; CDC *vs*. mMem; CDC *vs*. sMem; CDC *vs*. DN; mMem *vs*. sMem; mMem *vs*. DN; sMem *vs*. DN. A complete list of statistical comparisons can be found in **Supplemental Table S2**.

Finally, we asked if there was evidence for antigen selection in the post-CDC mutations using the BASELINe algorithm that detects and quantifies mutation selection using large datasets such as those generated here by NGS (**Figure 6**). Of note, the program extends the traditional approach based on replacement to silent ratio (R:S), refining it by considering the intrinsic mutational biases observed for SHM. The Bayesian approach used also quantifies the uncertainty for each estimate. Specifically, the program provides a distribution that measures “Selection Strength” for a set of sequences, together with an associated *P* value. The selection strength measure is analogous to the standard R:S ratio, with positive values indicating greater than expected R mutations, and negative values indicating fewer than expected R mutations. In an adaptive immune response, therefore, an increase in R:S would be expected in the VH CDRs, leading to a structural change in these regions and implying positive selection by antigen and a decreased R:S would be expected in the VH FRs, implying negative selection and hence avoidance of structural changes in the Ig as a whole. Thus, true underlying selection for antigen is reflected by a structurally intact Ig with positive selection strength values in CDRs and negative values in FRs.

Since the normal cellular equivalent of a CLL cell is still a matter of debate (28–31), we used a database containing IGHV-IGHD-IGHJ sequences from several normal B-cell types generated with the same methodology, i.e., memory B cells (isotype-switched, sMem, and IgM only, mMem), marginal zone B cells (MZ), and IgD^-^CD27^-^ (“double negative”, DN) B cells, as controls (32) (**Figure 6**). DN B lymphocytes are a memory population that exhibits IGHV mutations (33), albeit at a level less than classical memory cells, that are expanded in autoimmune settings (34) and upon aging (35).

As expected, both memory B cell subsets displayed significantly positive selection strength in the CDRs and significantly negative values in the FRs. The DN B cell subset exhibited similar findings. The extended group of CDC sequences showed a significantly reduced selection strength in the CDRs than memory and DN cells, with some evidence of negative selection (broadly, silent mutations outnumbering replacement mutations). This pattern resembled that of MZ B cells. However, the selection strength in the FRs was like the mMem, sMem, and DN subsets. In conspicuous contrast, there was a striking difference for the post-CDC mutations, both High and Low complexity, in that there was a markedly reduced and negative selection in the CDRs and positive selection strength for changes in the FRs. Finally, the IGHV-IGHD-IGHJ non-productive rearrangements bear the least evidence for VH CDR selection for mutations but a selection for VH FR mutations somewhat less than that for the post-CDC mutations.

Together, the R:S in the VH CDRs and FRs of the post-CDC mutations are categorically inconsistent with selection for enhanced antigen binding as a reason for the distribution of non-synonymous mutations.

### IGHV-IGHD-IGHJ Heterogeneity and Mutation Extent and Targeting Have Implications for Patient Clinical Courses

Since the presence or absence of significant numbers of IGHV gene mutations has proven to be an excellent prognostic indicator, we asked if intraclonal differences in IGHV-IGHD-IGHJ sequence measured throughout the entire rearrangement might provide additional information. For the 61 cases that displayed intraclonal heterogeneity, neither the percentage of intraclonal IGHV-IGHD-IGHJ variants nor the percentage of the PSCs, including or excluding the CDC, correlated significantly with time to first treatment (TTFT) (**Supplemental Figure S3**).

**Figure 7:**
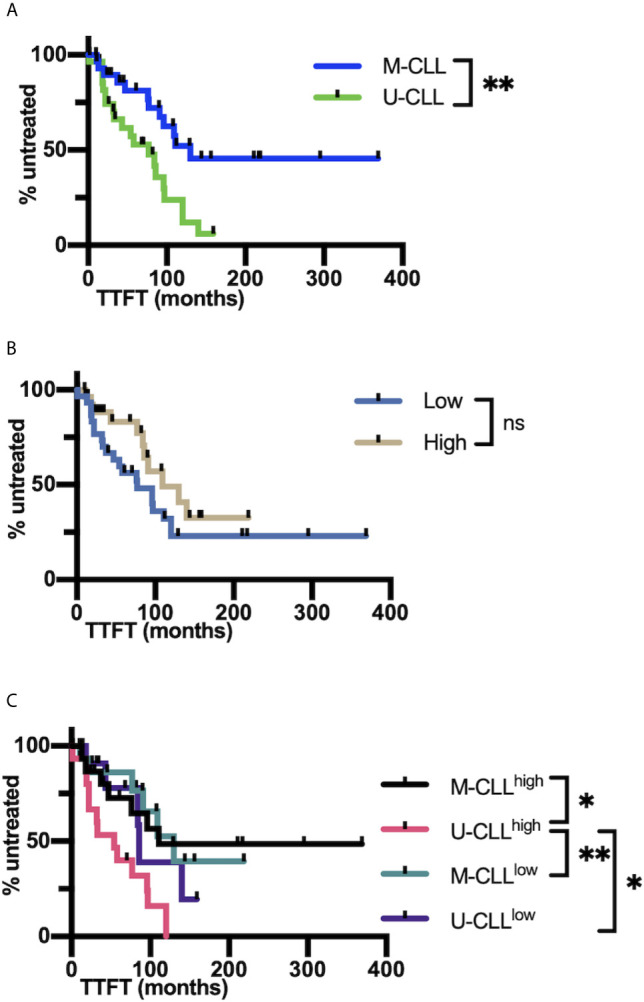
IGHV-D-J complexity correlates with clinical course based on time to first treatment. **(A)** Time to first treatment (TTFT) in months of the patients falling into the *IGHV*-mutated (M-CLL; blue) and *IGHV*-unmutated CLL subsets (U-CLL; green). **(B)** TTFT in months of the patients falling into the post-CDC complexity categories, Low and High. **(C)** TTFT (months) of the patients based on a combination of IGHV-mutation status plus the post-CDC complexity categories, U-CLL^High^, U-CLL^Low^, M-CLL^High^, and M-CLL^Low^. Statistics are displayed only when *P-value ≤*0.05 (*P-value ≤0.05, **P-value ≤0.01). NS, Not statistically significant.

When the clinical course (TTFT) of the cohort was analyzed using Cox regression, no significant interaction between mutation and complexity was observed (*P* = 0.22). Accordingly, when the interaction term was deleted from the model and the cases were divided into the U-CLL and M-CLL subgroups, there was, as expected a significant difference in TTFT between U-CLL and M-CLL (*P* = 0.0125; **Figure 7A**). However, when the cohort was divided based on IGHV-IGHD-IGHJ complexity (High and Low), TTFT was not different between the groups (*P* = 0.15; **Figure 7B**). Although we did not find evidence for a significant interaction between the IGHV-mutation groups and the complexity groups, those U-CLL patients in the High complexity group (U-CLL^High^) had a significantly shorter TTFT compared to both the M-CLL^Low^ (*P <*0.0102) and M-CLL^Hi^ (*P <*0.008) complexity groups (**Figure 7C**). Moreover, the estimates of the hazard ratios for U-CLL^High^ *vs*. U-CLL^Low^ and M-CLL^High^ *vs*. M-CLL^Low^ we were 2.59 and 1.02, respectively. This numerically suggests an interaction effect, i.e., that complexity is associated with TTFT in the U-CLL group but not in the M-CLL group). See **Supplemental Information** for details of the analyses.

Additionally, there was no significant mutation × complexity interaction (*P* = 0.7) for OS. Although the estimates of the hazard ratios for U-CLL^High^ *vs*. U-CLL^Low^ and M-CLL^High^ *vs*. M-CLL^Low^ appeared to differ numerically (0.40 and 0.62, respectively), the insignificant *P*-values, wide confidence intervals, and lack of pairwise differences between groups do not support a mutation × complexity interaction for OS.

## Discussion

We have examined the frequency and characteristics of intraclonal IGHV-IGHD-IGHJ diversification occurring in the progeny of the CLL clones defined in the clinic by Sanger sequencing. This has been a question of interest for several decades, and the answer that has slowly but progressively emerged as DNA sequencing technologies have become more sensitive and precise is that such diversification occurs. Using an NGS approach that achieved an average sequencing depth of 2,300,000× and allowed improved error correction due to the use of UMIs, we addressed the problem in 62 well characterized, untreated CLL patents. Our approach indicated that IGHV-IGHD-IGHJ sequence diversity occurs in virtually every CLL clone (~98%), with an average of 540 clonal variants observed per sample.

In addition to the surprising frequency at which intraclonal heterogeneity occurred, the size that the PSC can achieve in a given patient was also unexpected. When comparing the frequency of individual PSCs to the other intraclonal sequences (excluding the CDC), this reached 9% and was much larger (up to 88%) in some patients. Additionally, when using the absolute lymphocyte count in a patient’s blood at the time of sample collection to calculate numerical size, the PSC was almost always ≥10 cells/µl and could reach >3,600 cells/µl. In a healthy person, the latter level of clonal B cells would be consistent with monoclonal B-cell lymphocytosis (36), a documented pre-CLL disease (37). Moreover, the level of complexity of IGHV-IGHD-IGHJ intraclonal diversification was unanticipated, ranging from a single mutation to a series of mutations, often with several and occasionally multiple branch points.

Finally, the presence, frequency, and extent of post-transformation IGHV-IGHD-IGHJ mutations occurred equally in U-CLL and M-CLL. This indicates that the initial mutation load does not affect the ability of CLL clones to develop IGHV-IGHD-IGHJ diversity after leukemia develops. The finding also suggests strongly that the lack of mutations found in a U-CLL clone is not due to an inherent inability to carry out SHM. Rather, it strongly implies that the absence/scarcity of IGHV mutations in the normal B lymphocyte that converted to a CLL cell was dictated by signals delivered to the cell *via* the microenvironment prior to transformation.

These findings also suggested that when individual IGHV-IGHD-IGHJ subclones are found in larger numbers, exemplified by the PSCs, these cells bear a biologic advantage. We, therefore, analyzed the characteristics of the mutations occurring in the post-CDC setting, trying to define the mutation machinery responsible for the downstream mutations. We focused not only on mechanisms involved in targeting the IG loci, and thereby responsible for the generation of antigen-binding diversity in normal B lymphocytes, but also on mechanisms that could account for mutations found outside IG loci in CLL clones. The former included AID and error prone Polη, and the latter APOBEC and Signatures 1 and 5 in the COSMIC database. Although certain clones significantly expressed AID and/or Polη characteristics, none significantly exhibited signatures in the IGHV-IGHD-IGHJ region consistent with the actions of APOBEC or COSMIC Signatures 1 and 5. The approach also revealed that the percent of the post-CDC mutations within a given sample that could be assigned to the actions of AID and Polη was decreased very significantly when transitioning from the CDC to the post-CDC settings, suggesting that most of the mutations occurring post-leukemic transformation were not the consequences of an AID/Polη mechanism(s).

Because of these findings, we carried out similar analyses on the CLL sequences in the CDC set. Since not all the CDC samples bore IGHV somatic mutations (49 of 62), we also scrutinized a larger database of CLL clones comprised of 2,084 sequences. Both the CDC set directly related to this study and the expanded CLL set revealed the same features as the post-CDC mutations, with samples exhibiting significance for AID and Polη (AID > Polη), and none for the mutation mechanisms that affect non-IG loci in CLL. However, the percentage of mutations attributable to AID and Polη was very significantly higher in both CDC sets than for the post-CDC mutations, confirming the impression that the influence of AID/Polη mechanisms decreased substantially after leukemic transformation. When including comparisons of mutations detected in clonally expanded, non-productive IGHV-IGHD-IGHJ rearrangements, these mutations bore less evidence for AID and Poln actions than the CDC but more than the post-CDC mutations.

Since AID and Polη are essential and important components of SHM, respectively, we also investigated another feature of SHM, the ratio of mutations that represent transitions to transversions. In the normal SHM setting, the Ts : Tv is ~ 1.5:1. Notably, the Ts : Tv for all post-CDC mutations, productive or not, was considerably lower than this, and when the degree of mutational complexity was added to this analysis the High complexity samples had a more significantly lower Ts : Tv than the low complexity samples. Notably, when restricting the analysis to mutations bearing an AID targeting signature, the Ts : Tv was still below that expected for SHM.

Finally, we investigated if the post-CDC mutations were consistent with selection for improved antigen binding, based on comparing the expected with the observed R to S mutation ratio (R:S), using as controls normal mature B cell subsets as well as the non-productive IGHV-IGHD-IGHJ rearrangements identified. This indicated that, unlike the mutations found in memory and DN B cells and to a somewhat lesser extent in the starting CDC and MZ B cells samples, mutations in the productive post-CDC and non-productive IGHV-IGHD-IGHJ rearrangements showed a marked decrease of the R:S in CDRs and a significant increase in the R:S in FRs. These features are the opposite of what is expected for the selection of AID-mediated mutations that improve the affinity of antigen binding and for the preservation of an intact membrane IG and B-cell receptor signaling.

Collectively, the analyses of the type and targeting of the post-CDC mutations and the predicted consequences of these on selection for antigen reactivity suggest that many of these mutations do not reflect outcomes seen with typical, AID-based SHM that, in the normal setting, would be subsequently selected for improved antigen binding and associated adaptive protection. The findings also suggest that the mutations that develop after leukemic transformation differ, in multiple ways (inducing mechanism, motif targeting, Ts : Tv, and antigen binding), from those that occurred before/up to the time of transformation.

Thus, are those mutations occurring downstream of the transformation event the result of deranged SHM mediated by AID or of another process? If the former, the derangement might be the consequence of a direct effect on the leukemic B cell occurring upon transformation. Additionally, the discrepancy might reflect a change in microenvironmental influences in play up to the time of transformation compared to subsequently. Certainly, the lymphoid microenvironment in which the normal B lymphocyte that converted into a CLL cell developed and was shaped is different from the one to which the subclones were exposed after leukemic transformation. Simplistically, the former was normal, structurally and functionally, whereas the latter was likely dysregulated in both ways. This change is at least due to the expansion of the leukemic clone, resulting in major degradation of the architecture of lymph nodes (38), thereby allowing access of CLL subclones to different microenvironmental inputs. This would be especially relevant if the normal B lymphocyte precursors of CLL develop outside of normal germinal center structures (39) and if the influences delivered at extrafollicular sites and the responses to these are distinct from those in classical GCs. Studies in mice indicate that such differences are likely between extrafollicular and follicular sites (40). Additionally, the quality and level of these signals could be affected by the leukemic cells themselves. This has been documented in CLL for T cells (41) and follicular dendritic cells (42), key constituents of mutation initiation and subclonal variant selection. The consequences of these structural and function changes in lymphoid tissue might increase mutation frequency and decrease selection of mutation variants in a normal physiologic manner. In this scenario, mutations occurring in the IGHV-IGHD-IGHJ region would decrease the BCR’s influence on survival and growth of the intraclonal subclones, shifting the advantage to subclones developing mutations outside IG loci and leading to more dysregulated B-cell function and resulting in more aggressive disease.

Alternatively, the detected post-transformation mutations could, in part, be physiologic. In this case, the strict avoidance of replacement mutations in the CDRs and the deduced selection for replacement mutations in the FRs could provide the post-CDC subclones an alternative way to receive beneficial interactions, such as allowing superantigen binding to structurally altered FRs. In this regard, Staphylococcal superantigen can bind to FR3 (43) or to a discontinuous epitope created by FRs 1 and 3 and CDR2 (44). Similarly, HIV gp120 can bind IG through an unconventional binding site comprised of an intact hypervariable loop and alterations in the FRs (45). Additionally, FR mutations could potentially promote self-association (46) which can result in autonomous signaling (47), thereby promoting subclonal survival and expansion. In this situation, mutations occurring in the IGHV-IGHD-IGHJ region post-transformation would enhance the BCR’s ability to advance survival and growth of the intraclonal subclones. The apparent selection for replacement mutations in IGHV subregions that differ from those targeted by AID suggest that CLL cells when diversifying in an altered tissue microenvironment might employ a different mutational mechanism with distinct target sites.

## Data Availability Statement

The datasets presented in this study can be found in online repositories. The names of the repository/repositories and accession number(s) can be found below: NCBI BioProject; PRJNA673787.

## Ethics Statement

The studies involving human participants were reviewed and approved by Institutional Review Boards of Northwell Health and of Dana Farber Cancer Center. The patients/participants provided their written informed consent to participate in this study.

## Author Contributions

DB and NC conceived the project and designed the experimental approach. DB, CT, SV, and TM performed experiments and data analyses. MC supplied IGHV-IGHD-IGHJ sequences from normal human B cell subpopulations. JB, SK, SF, KRR, JK, JB, and SA provided clinical samples and clinical correlations. AM, FG, SB, MC, FM, and MS offered interpretations and conceptual insights. All authors contributed to the article and approved the submitted version.

## Funding

DB received funding from the European Union’s Horizon 2020 Research and Innovation Programme under the Marie Skłodowska-Curie grant agreement No 794075. JB was supported by funding from the NIH’s National Cancer Institute through the R01 grant CA 213442. NC and KRR thank the Karches Family, The Nash Family Foundation, The Marks Foundation, and the Jean Walton Fund for Leukemia, Lymphoma, and Myeloma Research for their support of the Feinstein Institutes’ CLL Research & Treatment Program. MS and TM are supported by a Multi-PI grant 1R01AI132507-01A1 from NIAID.

## Conflict of Interest

JB has served as a consultant for Abbvie, Acerta, Astra-Zeneca, Beigene, Catapult, Dynamo Therapeutics, Eli Lilly, Juno/Celgene/Bristol Myers Squibb, Kite, MEI Pharma, Nextcea, Novartis, Octapharma, Pfizer, Rigel, Sunesis, TG Therapeutics, and Verastem; received honoraria from Janssen; received research funding from Gilead, Loxo, Sun, TG Therapeutics, and Verastem; and served on data safety monitoring committees for Invectys. NC has received research funding from Verastem, Argenx, and Janssen.

The remaining authors declare that the research was conducted in the absence of any commercial or financial relationships that could be construed as a potential conflict of interest.
