# Supplementary material for: Post-Transformation IGHV-IGHD-IGHJ Mutations in Chronic Lymphocytic Leukemia B Cells: Implications for Mutational Mechanisms and Impact on Clinical Course
Source: Front Oncol. 2021 May 25;11:640731. doi: 10.3389/fonc.2021.640731 (PMC8186829; doi:10.3389/fonc.2021.640731)
Supplement: Supplementary file 1 [file DataSheet_1.docx]

**STATISTICAL METHODS**

We carried out a Cox proportional hazards regression analysis with mutation, complexity, and their interaction (mutation x complexity) in the model. The “interaction” between mutation and complexity measures whether the effect of complexity in the mutated group is the same or different from its effect in the unmutated group. To visualize these results, the Kaplan-Meier curves for TTFT and survival are provided for the four groups (M^High^, M^Low^, U^High^, U^Low^). Pairwise multiple comparisons were carried out for differences amongst the four groups without adjustment for multiple testing. All results were considered statistically significant if p<0.05.

**RESULTS**

**Time to First Treatment (TTFT)**. There was no significant mutation x complexity interaction (*P* = 0.2247); in other words, it could not be demonstrated that there was a differential effect of complexity between the mutated and unmutated groups. However, despite the lack of statistical significance, the *estimates* of the hazard ratios for M^High^ vs. M^Low^ and U^High^ vs. U^Low^, were 1.02 and 2.59, respectively, suggesting, numerically, an interaction effect (i.e. that complexity is associated with TTFT in the unmutated group but not in the mutated group). It should be noted that when performing the six pairwise comparisons amongst the four groups, TTFT was significantly longer for M^High^ vs. U^Low^ (*P* = 0.008) and for M^Low^ vs. U^High^ (*P* = 0.0102).

Analysis of main effects in this model failed to demonstrate an overall effect of mutation or complexity. (When the interaction effect is removed from the model, the TTFT was significantly longer for the mutated group (*P* = 0.0125) and there was no effect of complexity (*P* = 0.1510))

**Overall Survival**. There was no significant mutation x complexity interaction (*P* = 0.7200); in other words, it could not be demonstrated that there was a differential effect of complexity between the mutated and unmutated groups. Similar to what was observed in the TTFT analysis, the estimates of the hazard ratios for M^High^ vs. M^Low^ and U^High^ vs. U^Low^, appeared to differ numerically (0.40 and 0.62, respectively), but the very large p-values, wide confidence intervals and lack of pairwise differences between groups do not support any speculation as to a mutation x complexity interaction.

Like the TTFT result, analysis of main effects in this model failed to demonstrate an overall effect of mutation or complexity. (When the interaction effect is removed from the model, neither mutation nor complexity were significantly associated with survival (*P* = 0.078 and *P* = 0.243, respectively.).
