# Supplementary figures and images for: Post-Transformation IGHV-IGHD-IGHJ Mutations in Chronic Lymphocytic Leukemia B Cells: Implications for Mutational Mechanisms and Impact on Clinical Course
Source: Front Oncol. 2021 May 25;11:640731. doi: 10.3389/fonc.2021.640731 (PMC8186829; doi:10.3389/fonc.2021.640731)

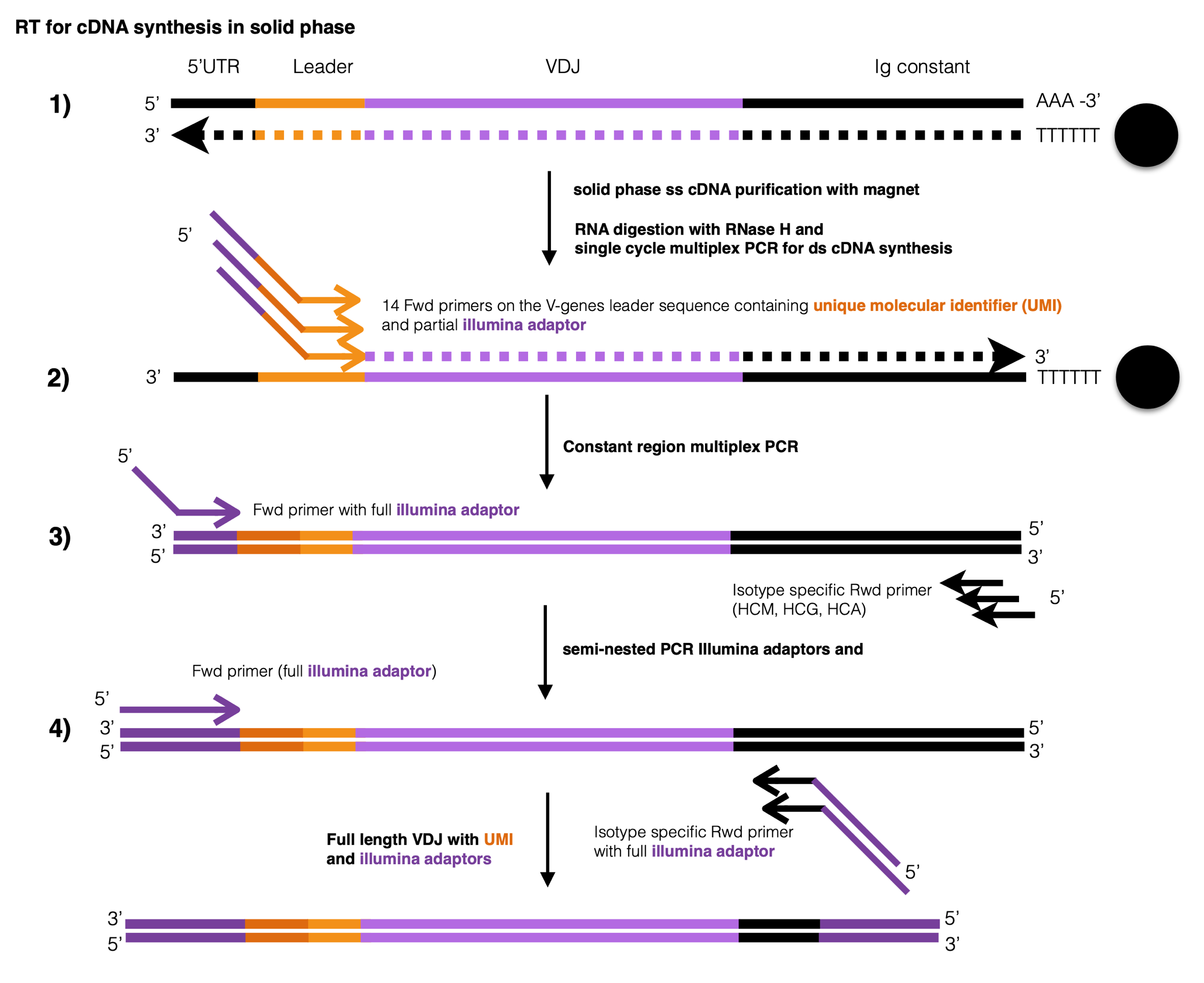

Supplement: Supplementary file 2 [file Image_1.tiff]

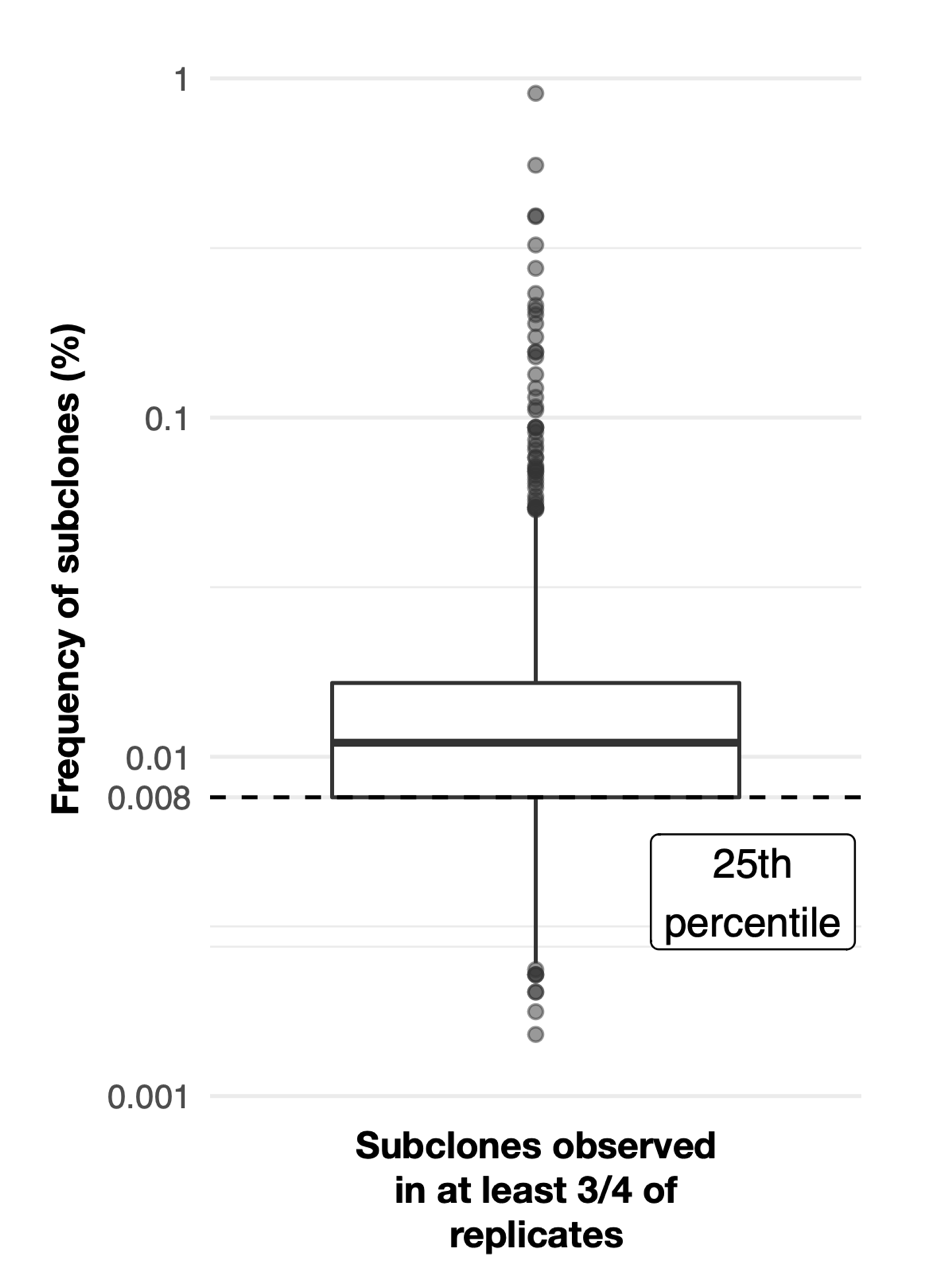

Supplement: Supplementary file 3 [file Image_2.tiff]

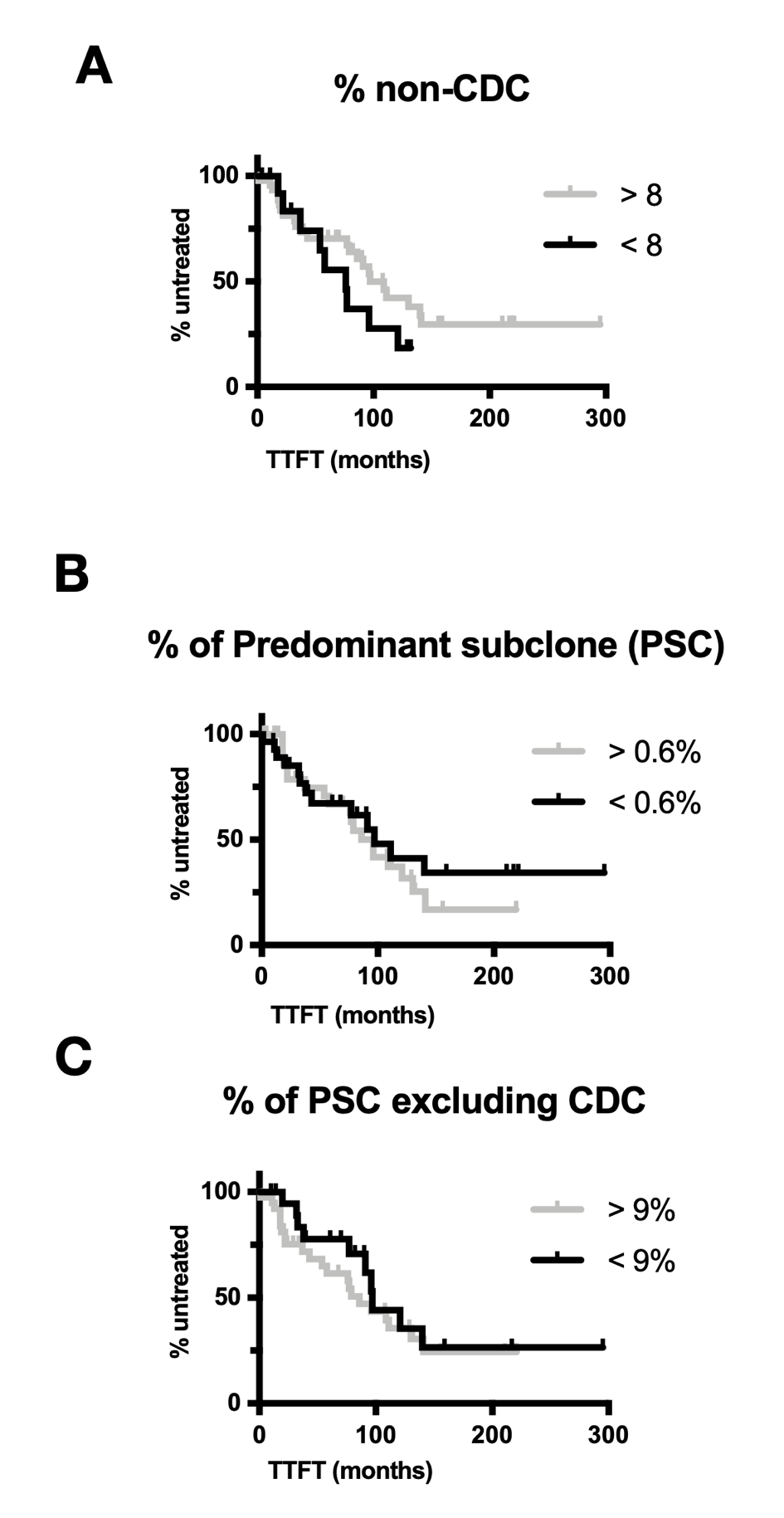

Supplement: Supplementary file 4 [file Image_3.tiff]
